# Supplementary material for: Solution conformational differences between conventional and CENP-A nucleosomes are accentuated by reversible deformation under high pressure
Source: Chromosome Res. 2025 Jun 12;33(1):11. doi: 10.1007/s10577-025-09769-z (PMC12159107; doi:10.1007/s10577-025-09769-z)
Supplement: Supplementary file 1 — Supplementary file1 (DOCX 3.35 MB) [file 10577_2025_9769_MOESM1_ESM.docx]

**Supplemental Materials**

**Solution conformational differences between conventional and CENP-A nucleosomes are accentuated by reversible deformation under high pressure**

Kushol Gupta^1^$^, Nikolina Sekulić^1,2*^$^, Praveen Kumar Allu^1^, Nicklas Sapp^1^, Qingqiu Huang^3^, Kathryn Sarachan^1#^, Mikkel Christensen^4^, Reidar Lund^4^, Susan Krueger^5,6^, Joseph E. Curtis^5^, Richard E. Gillilan^3^, Gregory D. Van Duyne^1^, Ben E. Black^1$^

^1^Department of Biochemistry & Biophysics, Perelman School of Medicine, University of Pennsylvania, Philadelphia, Pennsylvania 19104-6059, USA

^2^Norwegian Centre for Molecular Biosciences and Medicine, Faculty of Medicine, University of Oslo, Oslo, Norway

^3^Cornell High Energy Synchrotron Source, Cornell University, Ithaca, NY, 14853, USA

^4^Department of Chemistry, University of Oslo, Oslo, Norway

^5^Center for Neutron Research, National Institute of Standards and Technology, Gaithersburg, Maryland, 20899-6102, USA

^6^Department of Materials Science and Engineering, University of Maryland, College Park, Maryland 20742-2115, USA

^*^Present address: Department of Molecular Medicine, Institute of Basic Medical Sciences, Faculty of Medicine, University of Oslo, Oslo, Norway

#Present address: College of Arts & Sciences, Wilson College, Chambersburg, Pennsylvania, 17201, USA

^ co-first author

^$^ co-corresponding authors

Manuscript correspondence to:

Ben E. Black, Dept. of Biochemistry & Biophysics, Perelman School of Medicine at the University of Pennsylvania, 422 Curie Boulevard, Philadelphia, PA, USA 19104-6059 (Tel) 215-898-5039 Email: [blackbe@pennmedicine.upenn.edu](mailto:blackbe@pennmedicine.upenn.edu)

**Supplemental Methods**

**Rotating Anode.** Preliminary data were recorded on a Rigaku PSAXS small-angle X-ray scattering system equipped with Osmic mirror optics, a three-pinhole enclosed pre-flight path, an evacuated sample chamber with customized sample holder maintained at 4^o^C, and a gas-filled multi-wire detector. The instrument is served by a Rigaku MicroMax-007 HF microfocus rotating anode generator (Rigaku America, Woodland, T.X., U.S.A.). The forward scattering from the samples studied was recorded on a CCD detector and circularly averaged to yield one-dimensional intensity profiles as a function of *q* (*q*=4πsinθ/λ, where 2θ is the scattering angle, in units of Å^-1^). Data were reduced using SAXSGui v2.05.02 (Rigaku America) and matching buffers were subtracted to yield the final scattering profile. The sample-to-detector distance and beam center were calibrated using silver behenate and intensity converted to absolute units (cm^-1^) using a known polymer standard.

**Small-Angle X-ray Scattering at the Advanced Light Source Beamline 12.3.1 (SIBYLS).** Samples were centrifuged at 3,000 rpm for 10 min at 4°C prior to data collection. Data was collected using a 96-well plate handling sample robot, as previously described (Hura et al. 2009). All samples were characterized with 0.5, 1, and 6 s exposures at 20°C, at a wavelength of 1.0 Å. Data were automatically reduced using custom software to provide one-dimensional intensity profiles as a function of *q* (*q*=4πsinθ/λ, where 2θ is the scattering angle). Accessible scattering was recorded in the range of 0.010 < *q* < 0.35 Å^−1^.

**Small-angle Neutron Scattering at National Institutes of Standards and Technology Center for Neutron Research NG-3 (Glinka et al. 1998)**. Samples were prepared by dialysis at 4^o^C against matching buffers containing 20 mM potassium cacodylate pH 7.0, 5 mM EDTA and 0%, 20%, 70%, 80%, or 95% D_2_O for a minimum of three hours across membrane with 6-8kD cutoff (D-tube dialyzer (Novagen)). Samples were centrifuged at 10,000 *g* for 3 min at 4^o^C and then loaded into Hellma quartz cylindrical cells (outside diameter of 22 mm) with either 2-mm (for 95% and 80% D_2_O) and 1-mm pathlengths (70%, 20%, and 0% D_2_O) and maintained at 6°C. Sample concentrations for the SANS measurements were determined by Bradford analysis(Bradford 1976) and are shown in Table 2.

Scattered neutrons were detected with a 64 cm × 64 cm two-dimensional position-sensitive detector with 128 × 128 pixels at a resolution of 0.5 cm/pixel. Data reduction was performed using the NCNR Igor Pro macro package (Kline 2006). Raw counts were normalized to a common monitor count and corrected for empty cell counts, ambient room background counts and non-uniform detector response. Data were placed on an absolute scale by normalizing the scattered intensity to the incident beam flux. Finally, the data were radially-averaged to produce scattered intensity (*I(q)*, in cm^-1^) versus *q* (Å^-1^) profiles. The scattered intensities from the samples were further corrected for buffer scattering and incoherent scattering from hydrogen in the samples. Data collection times varied from 0.5 hour to 2 hours, depending on the instrument configuration, sample concentration and buffer conditions. Sample-to-detector distances of 11 m (*q*-range 0.006-0.043 Å^-1^, where *q*= 4πsin(θ)/λ, where λ is the neutron wavelength and 2θ is the scattering angle), 5 m (*q*-range 0.011–0.094 Å^−1^), and 1.5 m (detector offset by 20.00 cm, *q*-range 0.03–0.4 Å^−1^) were measured at a wavelength of 6 Å with wavelength spread of 0.15. We observed good agreement between R_g_ and I_0_ determined from inverse Fourier analysis using GNOM (Svergun 1992) and that determined by Guinier analysis. The program MuLCH (Whitten, Cai, and Trewhella 2008) was used to calculate theoretical contrast and to analyze contrast variation data.

**Small-angle Neutron Scattering (SANS) at Oak Ridge HFIR CG-3**. Experiments were conducted at the CG-3 BioSANS instrument at Oak Ridge National Laboratory (ORNL, Oak Ridge, TN). The wavelength of 6.0 Å with a wavelength spread of 0.15 Å was utilized at a 6 m sample-to-detector distance for one-hour exposures, providing an accessible *q* (where *q*=4πsinθ/λ, where 2θ is the scattering angle, in units of Å^-1^) of 0.008 < *q* < 0.15. Data were recorded at 6^o^C for all measurements in 1-mm (70%, 20%, and 0% D_2_O) or 2-mm (for 95% and 80% D_2_O) Hellma quartz cylindrical cells. To obtain normalized scattering intensities *I(q)* (cm^-1^) as a function of *q* (Å^-1^), empty cell and buffer cell scattering were subtracted from the sample scatter and normalized to absolute intensity units using a known polymer standard. Data reduction was performed using customized reduction scripts with the Mantid platform (Arnold et al. 2014). The program MuLCH (Whitten, Cai, and Trewhella 2008) was used to calculate theoretical contrast and to analyze contrast variation data. All scattering data were analyzed by the inverse Fourier transform using the program GNOM assessed by classical Guinier analyses.

**Supplemental Tables**

**Supplemental Table 1. Calculated Masses of Particles studied using Linear analysis of SANS I_0_.**

|  | Method 1 | | | Method 2 | |
| --- | --- | --- | --- | --- | --- |
| Sample | M_protein_ (kDa) | M_DNA_ (kDa) | Total M_Complex_ | M_protein_ (kDa) | Total M_Complex_ |
| CENP-A-601 | 131 ± 25 | 72 ± 23 | 203 ± 35 | 118 ± 19 | 207 ± 19 |
| H3-601 | 127 ± 12 | 86 ± 11 | 213 ± 16 | 125 ± 8 | 214 ± 8 |
| CENP-A-αSat | 77 ± 16 | 121 ± 20 | 198 ± 24 | 87 ± 16 | 176 ± 16 |
| H3-αsat | 76 ± 31 | 140 ± 39 | 216 ± 49 | 80 ± 11 | 169 ± 29 |

Method 1: M_protein_ and M_DNA_ were found by solving simultaneous equations using I_0_ values from 4-5 SANS contrast points. Concentrations were determined by Bradford assay.

Method 2: M_DNA_ was fixed and M_protein_ was determined using simultaneous equations.

**Supplemental Table 2. Parameters derived from Stuhrmann Analysis of SAXS/SANS data.**

| **Sample** | **R_c_ (Å)** | **α (cm^-1^)** | **β (cm^-2^)** | **Fit R^2^** |
| --- | --- | --- | --- | --- |
| **H3-601** | 39.0 ± 0.2 | 417.7 ± 81.2 | -698.6 ± 327.0 | 0.97 |
| *Hjelm 1977* | 40.5 | 450 |  |  |
| **CENP-A-601** | 39.9 ± 0.3 | 544.4 ± 153.5 | -320.1 ± 527.4 | 0.90 |
| **H3-αSat** | 40.9 ± 0.2 | 552.3 ± 159.4 | -596.0 ± 461.7 | 0.86 |
| **CENP-A-αSat** | 40.1 ± 0.4 | 305.9 ± 138.1 | -805.6 ± 452.6 | 0.97 |

**Supplemental Table 3**. **Calculated properties of proteins and DNAs used in this study.**

| Sample | Length | Molecular Weight (Daltons) | ν_bar_ (cm^3^/g) |
| --- | --- | --- | --- |
| human CENP-A | 140 a.a. | 15,991 | 0.741 |
| human Histone H3.1 | 135 a.a. | 15,248 | 0.744 |
| human Histone H4 | 103 a.a. | 11,367 | 0.747 |
| human Histone H2A | 134 a.a. | 14,420 | 0.749 |
| human Histone H2B | 126 a.a. | 13,906 | 0.744 |
| “601” DNA – Widom Strong Positioning DNA | 145 b.p. | 89,251 | 0.590 |
| α-satellite DNA | 145 b.p. | 89,166 | 0.590 |
| H3-601 |  | 199,573 | 0.675 |
| CENP-A-601 |  | 201,100 | 0.676 |
| H3-αSat |  | 199,173 | 0.675 |
| CENP-A-αSat |  | 200,648 | 0.675 |

**Supplemental Table 4. Global Core-Shell Cylinder Fitting of SAXS/SANS Data using SASVIEW (related to Supplemental Figure 5).**

| **Sample** | **Data** | **Fit Radius** | **Fit Length** | **χ^2^** |
| --- | --- | --- | --- | --- |
| H3-601 | Global Fit (5) | 19.5 Å ± 0.62 | 59.5 Å ± 2.8 | 2.8 |
|  | 0% D_2_O (SANS) |  |  | 1.6 |
|  | 20% D_2_O (SANS) |  |  | 2.4 |
|  | 80% D_2_O (SANS) |  |  | 0.9 |
|  | 90% D_2_O (SANS) |  |  | 1.1 |
|  | SAXS |  |  | 1.3 |
| CENP-A-601 | Global Fit (5) | 18.9 Å ± 0.12 | 70.3 Å ± 0.9 | 2.2 |
|  | 0% D_2_O (SANS) |  |  | 1.2 |
|  | 20% D_2_O (SANS) |  |  | 2.9 |
|  | 80% D_2_O (SANS) |  |  | 0.7 |
|  | 90% D_2_O (SANS) |  |  | 1.1 |
|  | SAXS |  |  | 0.6 |
| H3-αSat | Global Fit (6) | 18.9 Å ± 0.05 | 68.6 Å ± 0.3 | 3.7 |
|  | 0% D_2_O (SANS) |  |  | 1.1 |
|  | 20% D_2_O (SANS) |  |  | 0.9 |
|  | 70% D_2_O (SANS) |  |  | 3.4 |
|  | 80% D_2_O (SANS) |  |  | 2.5 |
|  | 90% D_2_O (SANS) |  |  | 2.8 |
|  | SAXS |  |  | 1.6 |
| CENP-A-αSat | Global Fit (6) | 18.1 Å ± 0.04 | 74.3 Å ± 0.2 | 2.6 |
|  | 0% D_2_O (SANS) |  |  | 1.1 |
|  | 20% D_2_O (SANS) |  |  | 0.8 |
|  | 70% D_2_O (SANS) |  |  | 1.5 |
|  | 80% D_2_O (SANS) |  |  | 0.9 |
|  | 90% D_2_O (SANS) |  |  | 0.9 |
|  | SAXS |  |  | 1.6 |

**Supplemental Table 5. Parameters derived from Analytical Ultracentrifugation**

| Sample | Sedimentation Equilibrium (SE)(Daltons) | |
| --- | --- | --- |
|  | 25^o^C | 4^o^C |
| H3-601 | 202,787 ± 1,437 | 187,233 ± 1,182 |
| H3-αSat | 199,226 ± 1,345 | 208,036 ± 1,316 |
| CENP-A-αSat | 197,476 ± 4,887 | 193,466 ± 4,339 |

SE-AUC experiments were carried out at 26,000 RPM in 20 mM Tris-HCl pH 7.5, 1 mM EDTA, and 1 mM DTT

Masses derived from SE experiments were calculated by global fitting of three concentrations of particle at three different speeds (7K, 9K, and 11Krpm).

**Supplemental Figures**


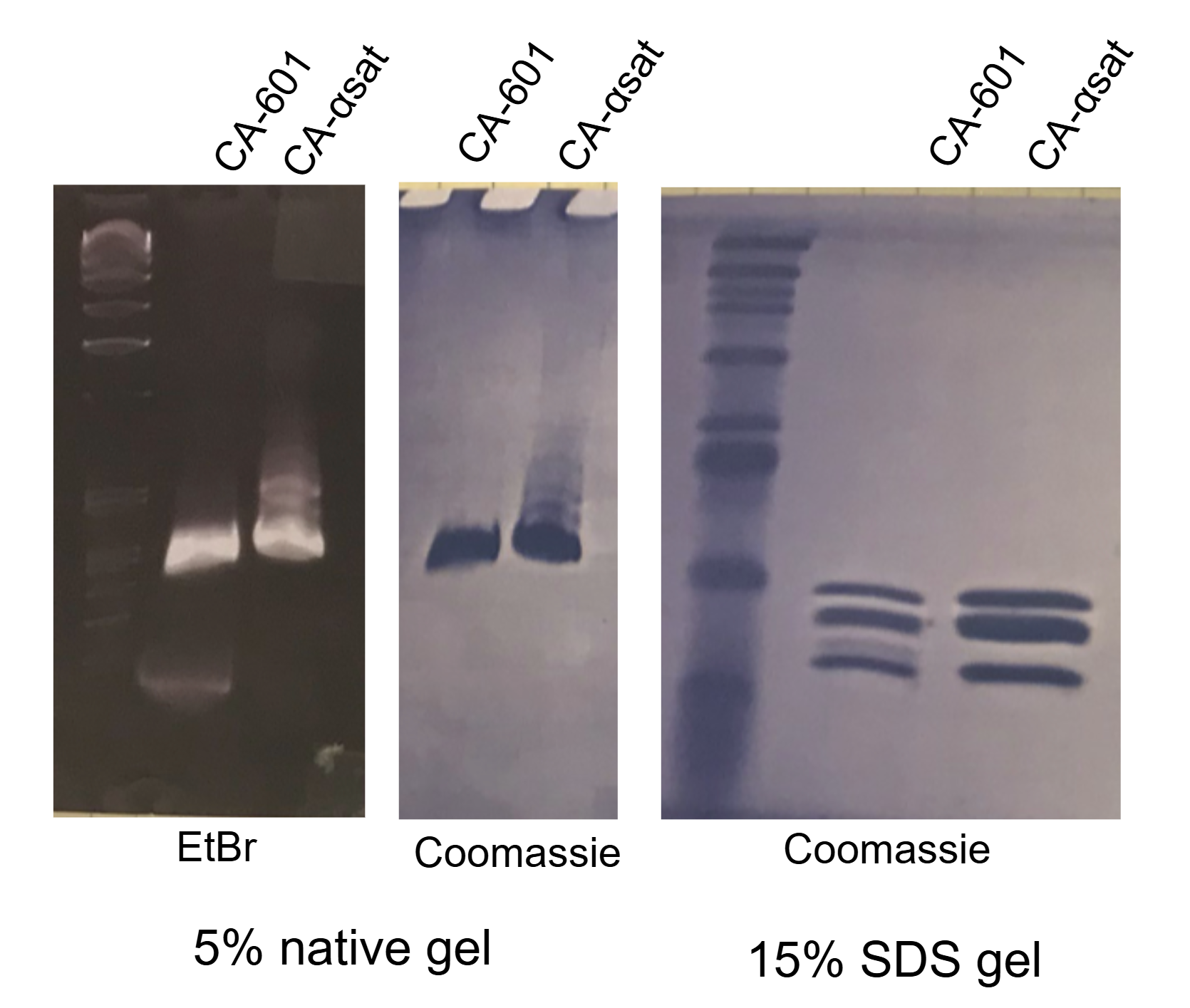


**Supplemental Figure 1. Representative PAGE analysis of reconstituted mononucleosomes.** Shown on the left are CENP-A nucleosomes prepared with either 601 or αSat DNA and separated on a 5% native PAGE. The DNA was visualized with ethidium bromide and protein with Coomassie. The same particles were analyzed by denaturing 15% SDS-PAGE (right), showing the presence of four histone proteins.

**
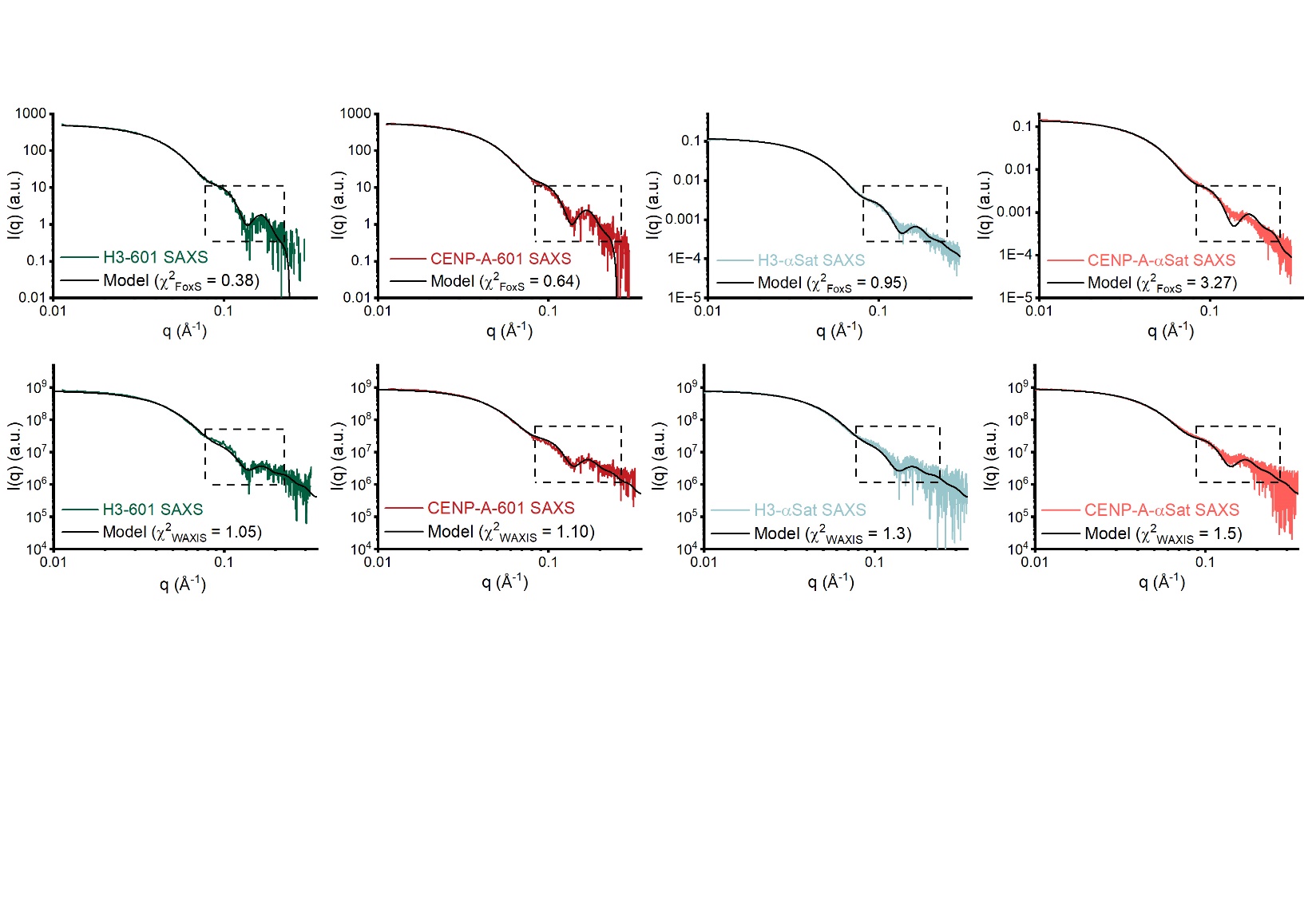
**

**Supplemental Figure 2. FoxS and WAXIS fitting of atomic models to SAXS Data.** Shown are the respective model fits (black lines) to SAXS data performed using FoxS (upper panels, implicit solvent boundary model) or WAXiS (lower panels, explicit solvent boundary calculation) for H3-601 (green), CENP-A-601 (red), H3-αSat (cyan), and CENP-A-αSat (light red). Shown in black boxes are middle-q regions of the fit most discrepant in the fitting, with a χ^2^ for the fit provided in the respective graph legends.

**
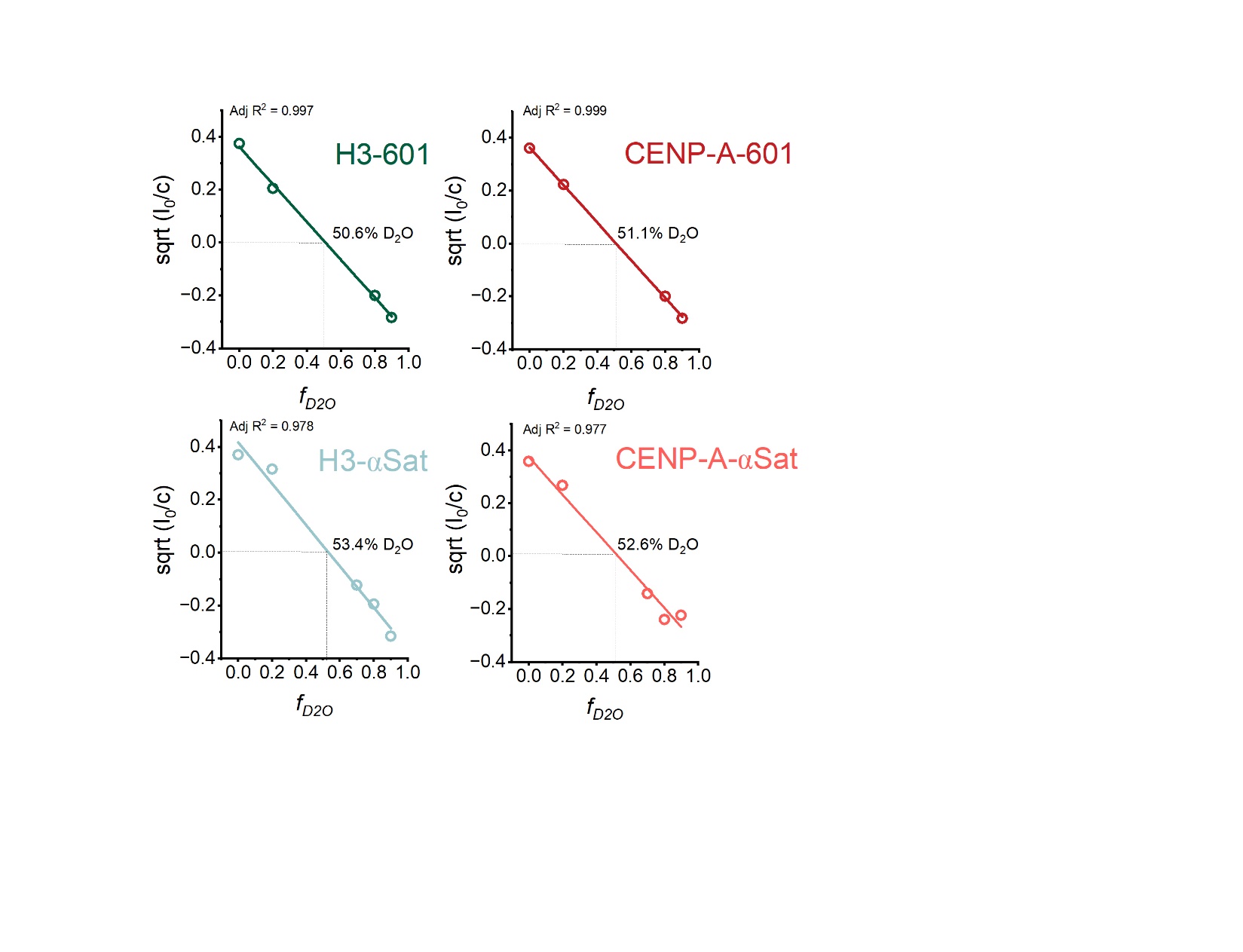
**

**Supplemental Figure 3.** **Zero-Angle Scattering from SANS.** The contrast-dependence of the zero-angle scattering is shown. The square root of the extrapolated zero angle scatter (I_0_, in absolute units of cm^-1^) divided by concentration (c) in mg/ml, plotted against the fractional D_2_O (*f_D2O_*). The fractional D_2_O can be directly related to the solvent scattering length density for each contrast. A linear plot in all four cases indicates monodispersity in these preparations (Stuhrmann and Duee 1975) and the least squares fit provides the overall contrast point where intensity is zero.


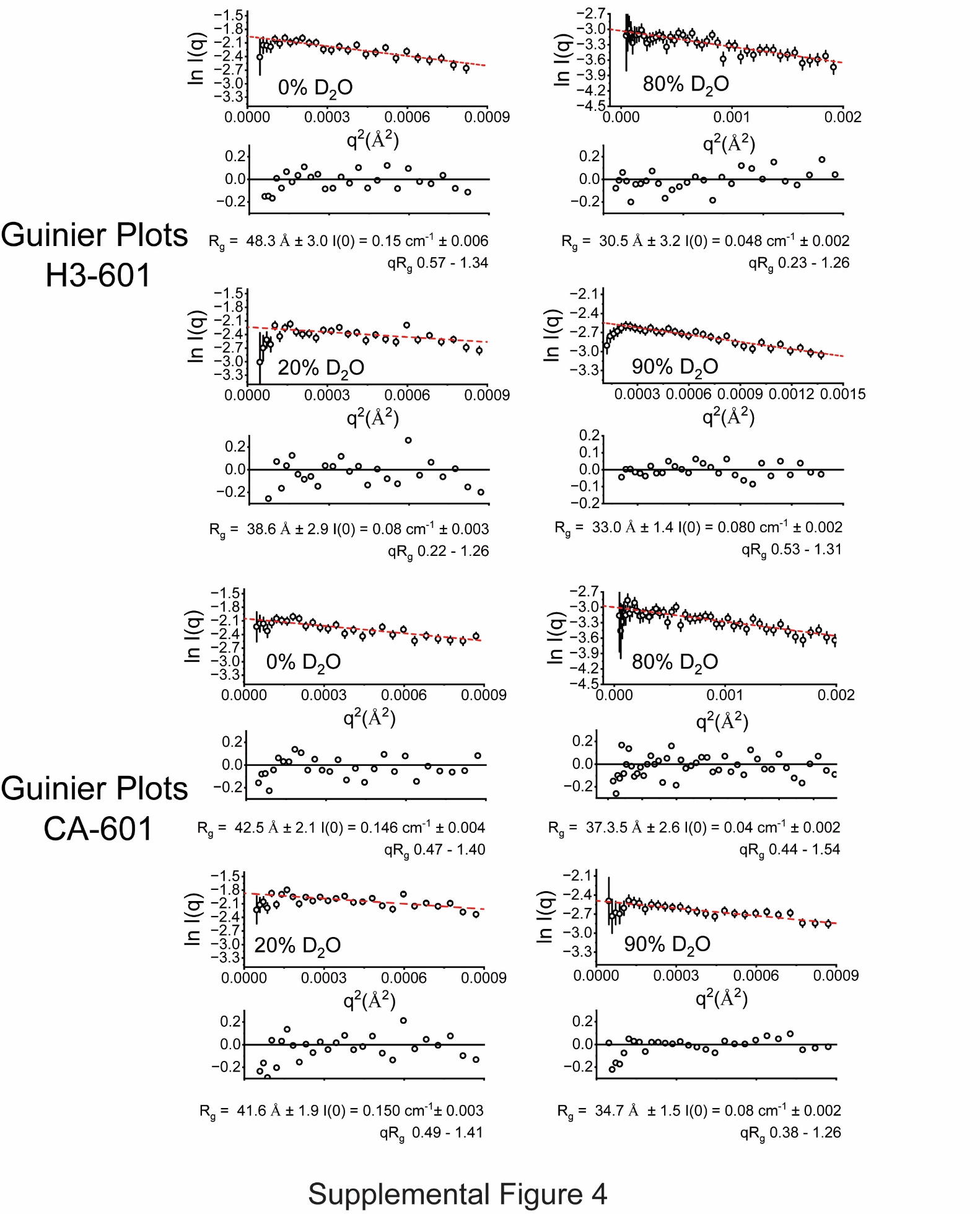


**Supplemental Figure 4. SANS Guinier Plots for H3-601 and CENP-A-601.** Guinier plot analyses (ln (I) vs. q^2^) of SAXS data (black open circles) for H3-601 and CENP-A601 NCPs, with residuals from the fitted lines shown below. Monodispersity is evidenced by linearity in the Guinier region of the scattering data and agreement of the I_0_ and R_g_ values determined with inverse Fourier transform analysis by the programs GNOM (Table 2). Guinier analyses were performed where qR_g_ ≤ 1.4.

**
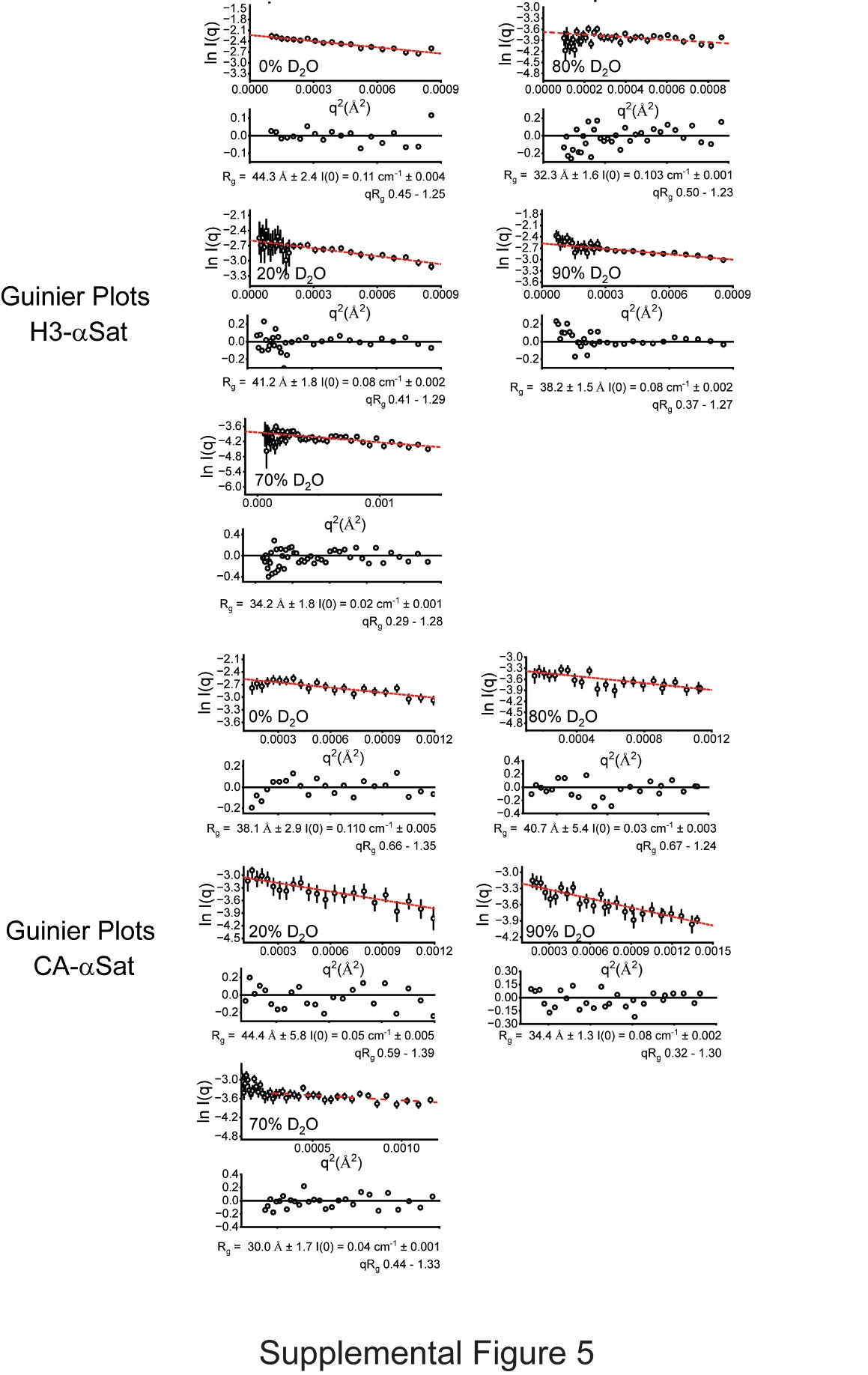
**

**Supplemental Figure 5. SANS Guinier Plots for H3-αSat and CENP-A-αSat.** Guinier plot analyses (red lines) (ln (I) vs. *q*^2^) of SAXS data (black open circles) for H3-αSat and CENP-A-αSat NCPs, with residuals from the fitted lines shown below. Monodispersity is evidenced by linearity in the Guinier region of the scattering data and agreement of the I_0_ and R_g_ values determined with inverse Fourier transform analysis by the programs GNOM (Table 1). Guinier analyses were performed where qR_g_ ≤ 1.4.

**
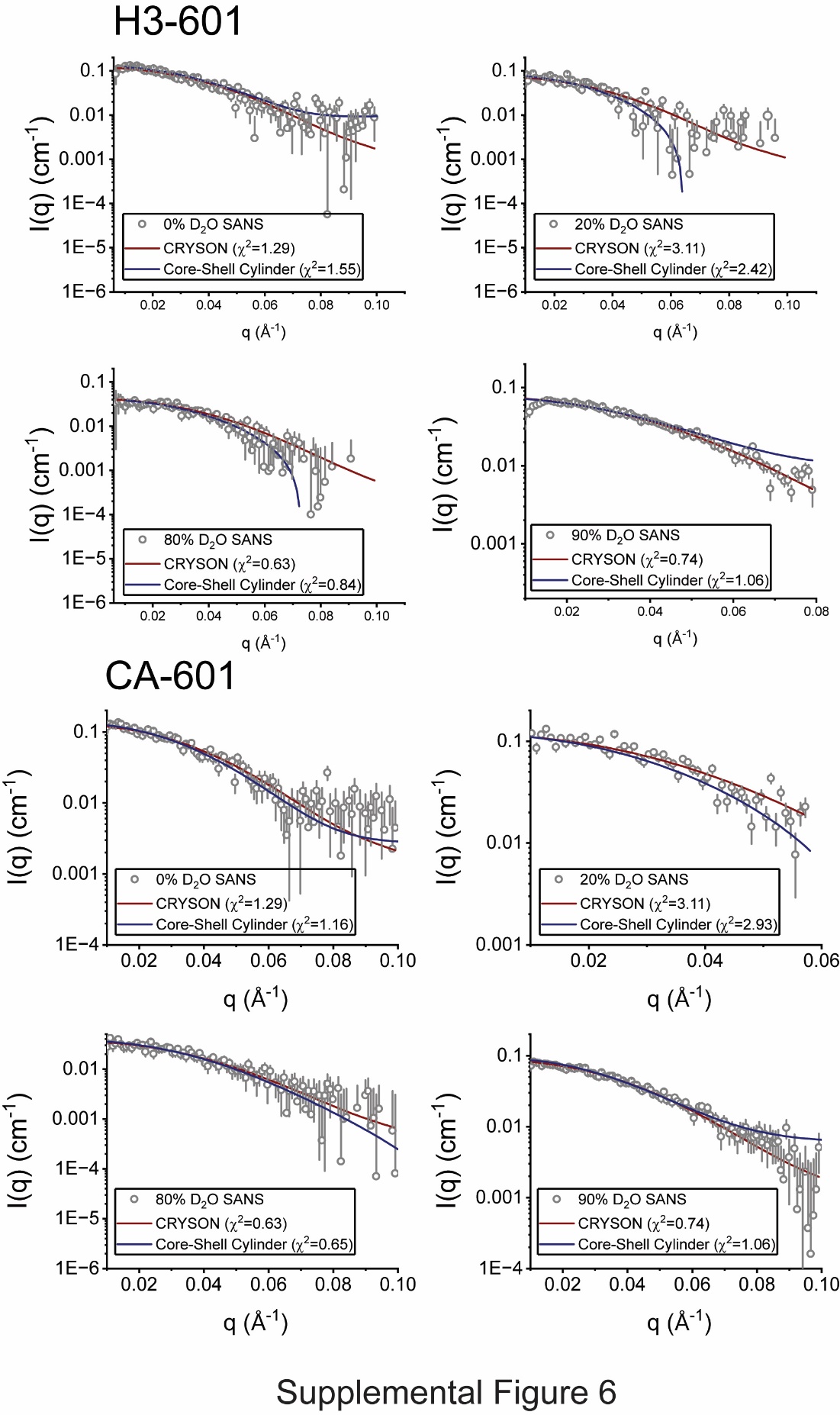
**

**Supplemental Figure 6. CRYSON and Core-Shell Ellipsoid Fitting of SANS Data for H3-601 and CENP-A-601.** Shown are individual CRYSON fits (red lines) of the H3-601 or CENP-A-601 atomic models to experimental SANS data for both H3- and CENP-A 601 particles at each of four different D_2_O concentrations, with the χ^2^ for each respective fit provided in the graph legend. Fits were performed where q_max_ < 0.1 Å^-1^. Shown as blue lines are the global core-shell ellipsoid fits for SANS data for either the H3-601 or CENP-A-601 particles, as implemented in the program SASVIEW. See **Supplemental Table 4** for the structural parameters derived and individual and global χ^2^ associated with this fitting. Error bars on the scattering data (grey circles) represent the uncertainty associated with the intensity recorded.

**
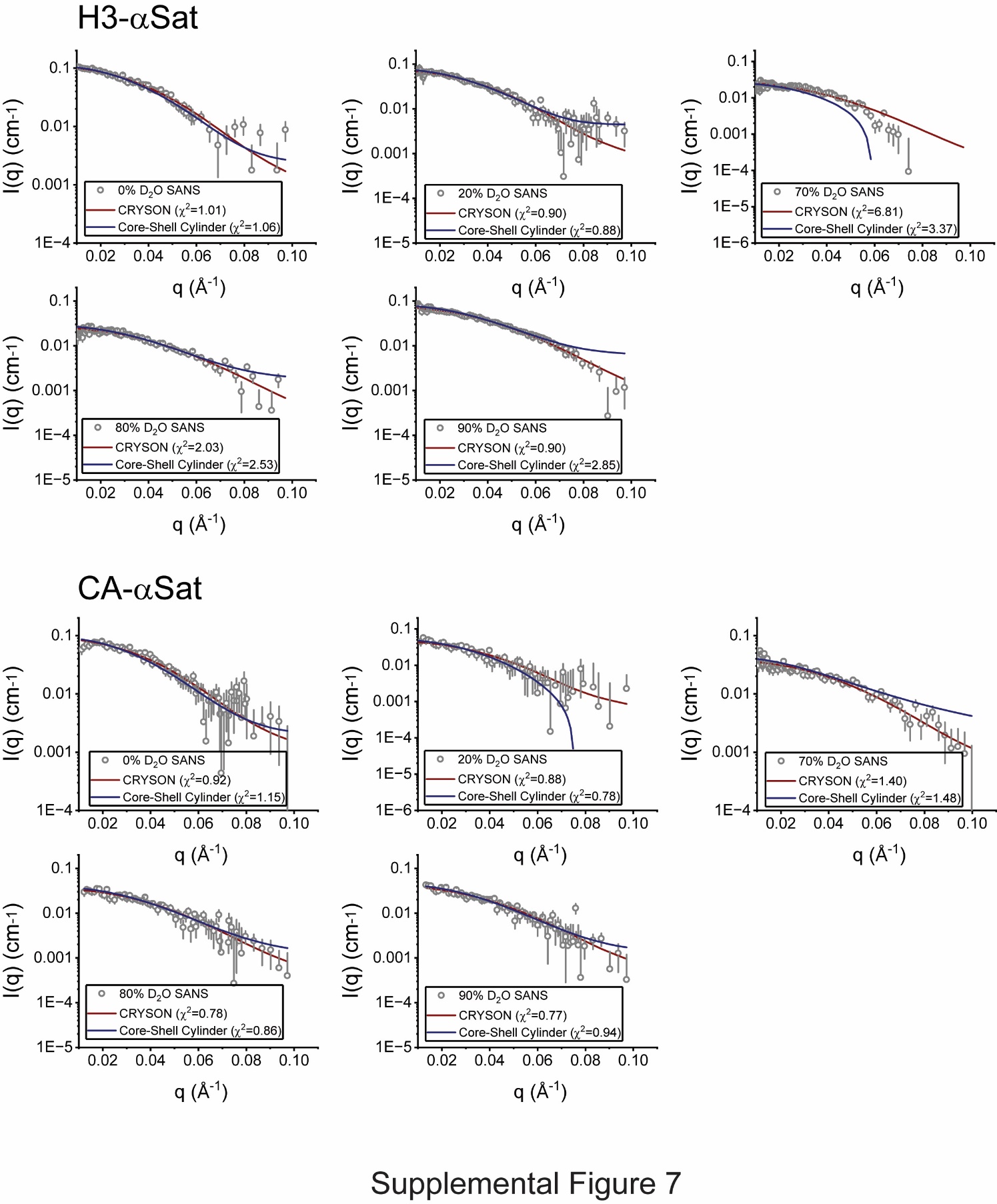
**

**Supplemental Figure 7. CRYSON and Core-Shell Ellipsoid Fitting of SANS Data for H3-αSat and CENP-A-αSat.** Shown are individual CRYSON fits (red lines) of the H3-601 or CENP-A-601 atomic models to experimental SANS data for both H3- and CENP-A αSat particles at each of five different D_2_O concentrations, with the χ^2^ for each respective fit provided in the graph legend. Fits were performed where q_max_ < 0.1 Å^-1^. Shown as blue lines are the global core-shell ellipsoid fits for SANS data for either the H3-αSat or CENP-A-αSat particles, as implemented in the program SASVIEW. See **Supplemental Table 4** for the structural parameters derived and individual and global χ^2^ associated with this fitting. Error bars on the scattering data (grey circles) represent the uncertainty associated with the intensity recorded.


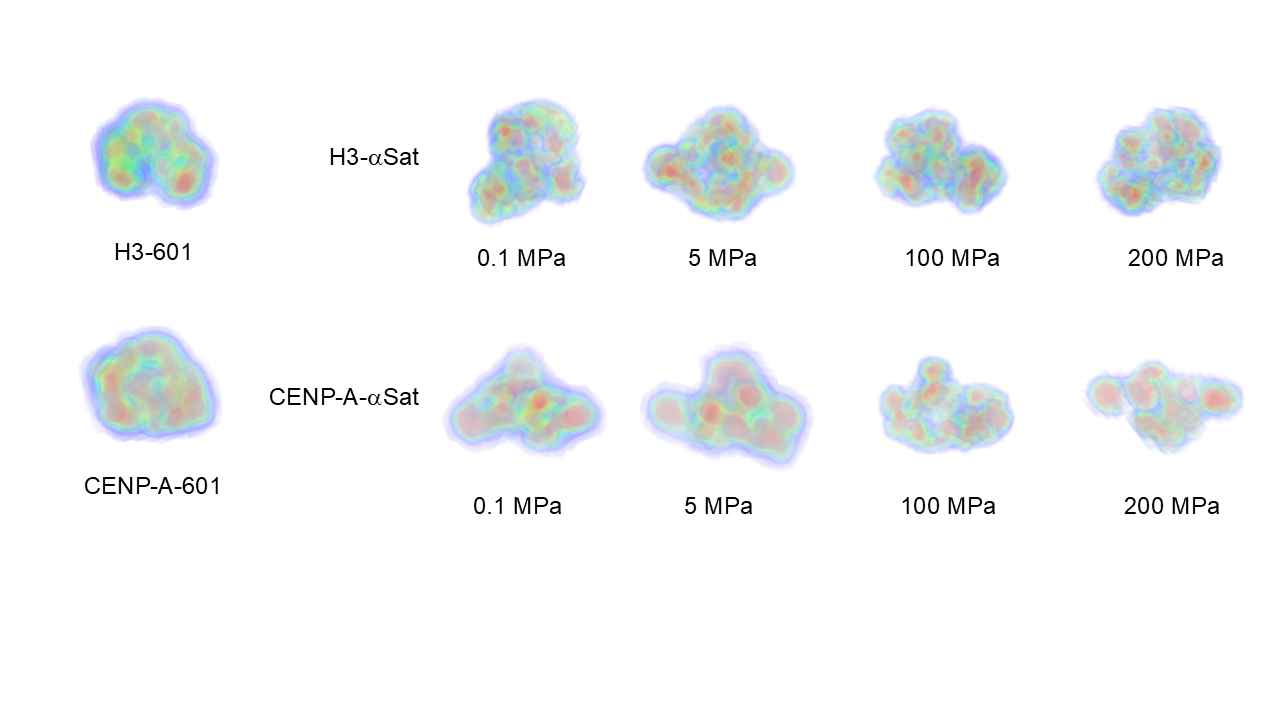


**Supplemental Figure 8. DENSS Reconstructions.** Shown are the DENNS reconstructions provided in **Figures 1,3, & 4**, but without any atomic structures docked. Electron density is colored with five contour levels of density rendered with these respective colors: 15σ (red), 10σ (green), 5σ (cyan), 2.5σ (blue), and -0.7σ (blue). The sigma (σ) level denotes the standard deviation above the average electron density value of the generated volume.

**References**

Arnold, O., J. C. Bilheux, J. M. Borreguero, A. Buts, S. I. Campbell, L. Chapon, M. Doucet, N. Draper, R. Ferraz Leal, M. A. Gigg, V. E. Lynch, A. Markvardsen, D. J. Mikkelson, R. L. Mikkelson, R. Miller, K. Palmen, P. Parker, G. Passos, T. G. Perring, P. F. Peterson, S. Ren, M. A. Reuter, A. T. Savici, J. W. Taylor, R. J. Taylor, R. Tolchenov, W. Zhou, and J. Zikovsky. 2014. 'Mantid—Data analysis and visualization package for neutron scattering and μ SR experiments', *Nuclear Instruments and Methods in Physics Research Section A: Accelerators, Spectrometers, Detectors and Associated Equipment*, 764: 156-66 <https://doi.org/10.1016/j.nima.2014.07.029>.

Bradford, Marion M. 1976. 'A rapid and sensitive method for the quantitation of microgram quantities of protein utilizing the principle of protein-dye binding', *Analytical Biochemistry*, 72: 248-54 <https://doi.org/10.1016/0003-2697(76)90527-3>.

Glinka, C. J., J. G. Barker, B. Hammouda, S. Krueger, J. J. Moyer, and W. J. Orts. 1998. 'The 30 m small-angle neutron scattering instruments at the National Institute of Standards and Technology', *Journal of Applied Crystallography*, 31: 430-45 Doi 10.1107/S0021889897017020.

Hura, G. L., A. L. Menon, M. Hammel, R. P. Rambo, F. L. Poole, 2nd, S. E. Tsutakawa, F. E. Jenney, Jr., S. Classen, K. A. Frankel, R. C. Hopkins, S. J. Yang, J. W. Scott, B. D. Dillard, M. W. Adams, and J. A. Tainer. 2009. 'Robust, high-throughput solution structural analyses by small angle X-ray scattering (SAXS)', *Nat Methods*, 6: 606-12 10.1038/nmeth.1353.

Kline, S. R. 2006. 'Reduction and analysis of SANS and USANS data using IGOR Pro', *Journal of Applied Crystallography*, 39: 895-900 10.1107/S0021889806035059.

Stuhrmann, H. B., and E. D. Duee. 1975. 'The determination of the scattering density distribution of polydisperse solutions by contrast variation: a neutron scattering study of ferritin', *Journal of Applied Crystallography*, 8: 538-42 doi:10.1107/S0021889875011211.

Svergun, D. 1992. 'Determination of the regularization parameter in indirect-transform methods using perceptual criteria', *Journal of Applied Crystallography*, 25: 495-503 doi:10.1107/S0021889892001663.

Whitten, A. E., S. Z. Cai, and J. Trewhella. 2008. ':: modules for the analysis of small-angle neutron contrast variation data from biomolecular assemblies', *Journal of Applied Crystallography*, 41: 222-26 10.1107/S0021889807055136.
